# Quality assurance using a photodiode array

**DOI:** 10.1120/jacmp.v12i2.3358

**Published:** 2011-01-31

**Authors:** M.J. Balderson, D.P. Spencer, I. Nygren, D.W. Brown

**Affiliations:** ^1^ Department of Physics and Astronomy University of Calgary Calgary Alberta Canada; ^2^ Department of Medical Physics Tom Baker Cancer Centre Calgary AB Canada

**Keywords:** quality assurance, photo diode, radiation light congruence

## Abstract

Improved treatment techniques in radiation therapy provide incentive to reduce treatment margins, thereby increasing the necessity for more accurate geometrical setup of the linear accelerator and accompanying components. In the present paper, we describe the development of a novel device that enables precise and automated measurement of geometric parameters for the purpose of improving initial setup accuracy, and for standardizing repeated quality control activities. The device consists of a silicon photodiode array, an evaluation board, a data acquisition card, and a laptop. Measurements that demonstrate the utility of the device are also presented. Using the device, we show that the radiation light field congruence for both 6 and 15 MV beams is within 1.3 mm. The maximum measured disagreement between radiation field edges and light field edges was 1.290±0.002 mm, while the smallest disagreement between the light field and radiation field edge was 0.016±0.003 mm. Because measurements are automated, ambiguities resulting from interobserver variability are removed, greatly improving the reproducibility of measurements across observers. We expect the device to find use in consistency measurements on linear accelerators used for stereotactic radiosurgery, during the commissioning of new linear accelerators, or as an alternative to film or other commercially available devices for performing monthly or annual quality control checks.

PACS numbers: 87.55.Qr, 87.56.Fc, 87.57.N‐, 87.15mn, 87.15mq

## I. INTRODUCTION

Over half of cancer patients receive some form of radiotherapy for treatment,^(1)^ and it is reasonable for patients to expect that their treatment be delivered as accurately as possible. Many factors affect the accuracy of a delivered treatment, including setup errors during the simulation process, patient setup errors during treatment, uncertainties in dose calculation, and errors that result from incorrect calibration and geometric setup of the linear accelerator. Numerous discussions of such errors have been previously presented in the literature.^(^ ^1^ ^–^ ^7^ ^)^ Among them, geometric accuracy of the components that position the patient and that produce and shape the radiation beam, play a limiting role in determining the overall accuracy of the patient treatment. This is especially true for cases of stereotactic radiosurgery, where a single dose of radiation is delivered to a lesion using highly conformal radiation beams.^(^ ^8^ ^–^ ^10^ ^)^ With the advent of newer technologies such as image guided radiotherapy, intensity‐modulated radiation therapy, and volumetric‐modulated arc therapy (VMAT), there is increased incentive to reduce treatment margins. Highly conformal radiation beams spare normal tissue while enabling increased tumor doses, but they also increase the likelihood and severity of geometric misses, and thus increase the necessity for improved geometric setup of the linear accelerator and associated components.

Standards and guidelines regarding geometric quality control have been presented by various professional organizations including the American Association of Physicists in Medicine (AAPM) and the Canadian Organization of Medical Physics (COMP).^(^ ^2^ ^,^ ^3^ ^)^ Geometric controls are most commonly performed using film, devices such as the PROFILER2 (Sun Nuclear, Melbourne, FL) and, in the case of stereotactic radiosurgery, using the Winston‐Lutz test.^(^ ^11^ ^,^ ^12^ ^)^ While these techniques have proven adequate in most situations, a major disadvantage common to all of them is that they are user‐dependent, making the results subjective. For example, using the PROFILER2 the user manually adjusts the edge of the light field to correspond to markers on the surface of the device. The edge is slightly blurred due to the penumbra of the light field, so different users will choose different edges from within the penumbra region of the light field.

In an effort to standardize geometric measurements over time and across observers, we have developed a novel device based on a photodiode array. The detector enables measurement of geometric parameters such as radiation light field congruence, and jaw movement reproducibility. Measurements and analyses are automated, which removes subjective error, and the small center‐to‐center distance between diodes in the photodiode array enables extremely precise measurements. In the current paper, we describe the design of the measurement device, outline the data analysis methods, and demonstrate the utility of the device by measuring the collimator angle and energy dependence of radiation light field congruence, as well as the reproducibility of mechanical and digital jaw movements. A comparison of the variability of repeated measurements made using the device, a common film technique, and the PROFILER2 is also presented.

## II. MATERIALS AND METHODS

### A. The detector

The detector is composed of a RadEye1 (Rad‐icon Imaging Corporation, Sunnyvale, CA, USA)^(13)^ silicon photodiode array which is connected to a National Instruments data acquisition card (DAQCard, National Instruments, Austin, TX) by means of an evaluation board. The evaluation board gives the user full software control over the RadEye1 image sensor and allows the user to acquire and save images for further analysis. The DAQCard is connected to a computer and the detector is controlled using LabVIEW software (National Instruments, Austin, TX) and routines provided by Rad‐Icon Imaging Corporation. The detector has an active area of 24.6 mm by 49.2 mm consisting of a 512 by 1024 matrix of silicon photodiodes on 48 μm centers.

**Figure 1:**
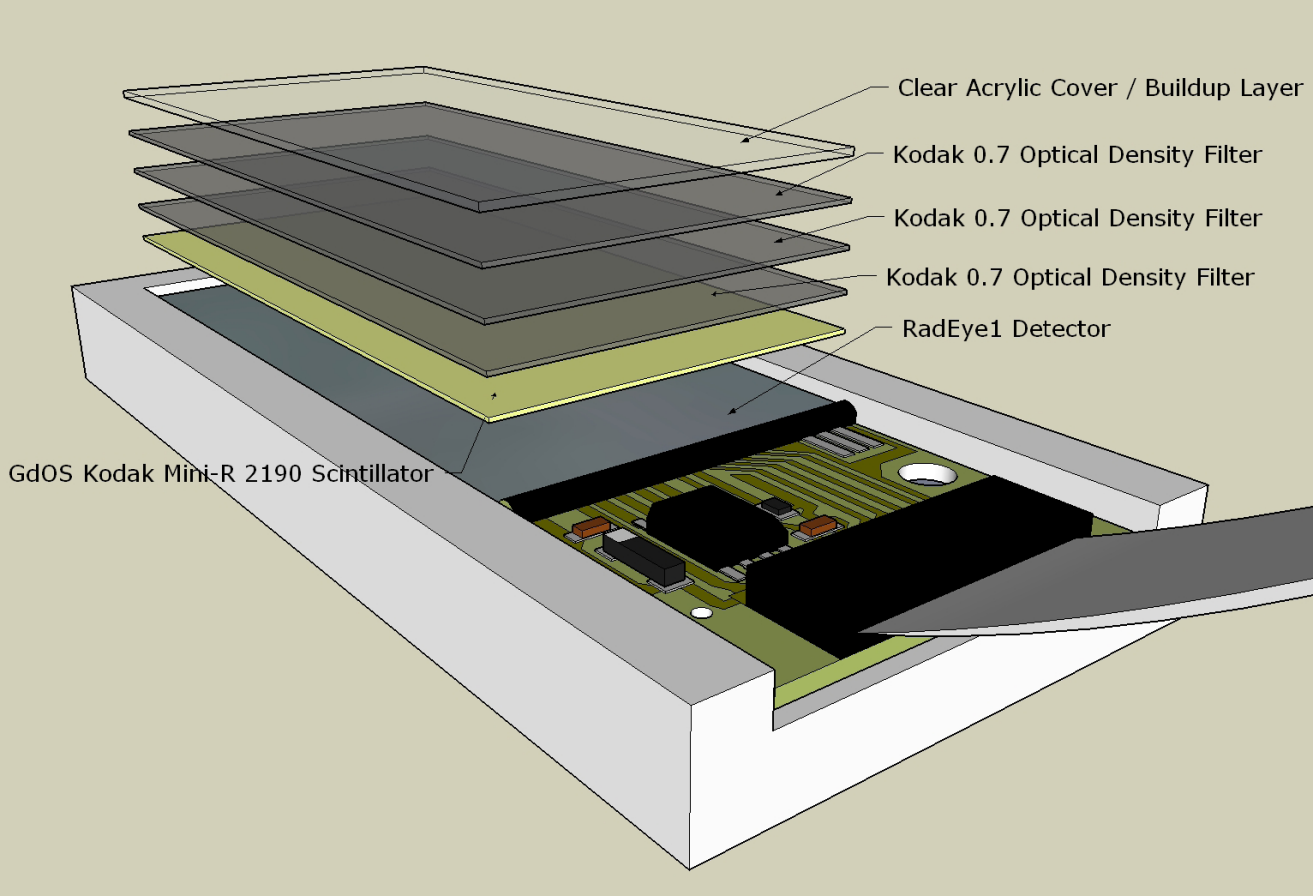
Schematic of the detector showing the photodiode array, GdOS Kodak Mini‐R 2190 scintillator, Kodak optical density filters, and the clear acrylic cap.

Figure 1 shows the design of the device. The RadEye1 detector is designed to detect both visible light and kV X‐rays. However, without modification, the detector is too sensitive to the light field of the linear accelerator and not sensitive enough to MV X‐rays. In order to increase the sensitivity to MV X‐rays, a 0.3 mm GdOS Kodak Mini‐R 2190 scintillator (Eastman Kodak Company, Rochester, NY) was placed on the surface of the photodiode array. In order to decrease sensitivity to the light field, three layers of 0.1 mm thick 0.7 OD Kodak optical density filters were added to the surface of the scintillator. A 1.5 mm layer of clear acrylic plastic was placed on top of the other layers to hold the other materials in place and to act as a buildup layer that further increased the signal produced by MV X‐rays. The buildup layer might have a small effect on the point spread function as the scatter from the buildup layer may cause blurring of the field edge. However, this blurring of the field edge will have minimal effect on our calculated field edge because scattering is generally isotropic, so the effect in the area of interest (50%) should be fairly uniform. In this configuration, the detector can be used to measure both 6 and 15 MV X‐rays as well as visible light, without having to add or remove anything from the device and without having to move the device in any way. This device is an improved version of that described by Nygren and Spencer^(14)^ where they show that the photodiode device is capable of measuring other machine parameters such as gantry sag and head tilt. Other machine parameters where this device might find use would be in the evaluation of the position of the multileaf collimator (MLC).

### B. Radiation light congruence at different energies

Radiation light field congruence measurements were acquired on a Varian Clinac 21EX linear accelerator (Varian Medical Systems, Palo Alto, CA). The gantry was leveled using a bubble level and all measurements were acquired at a gantry angle of 0° using a dose rate of 600 Monitor Units per minute (MU/min). In order to remove background, background images were acquired prior to each step in the procedure and subtracted from acquired images prior to analysis. The detector was positioned using the room lasers such that the surface of the detector was at 100 cm from the radiation source (100 cm SSD). The detector was aligned such that the length of the detector was in the radial direction and the width of the detector was in the transverse direction. Using the hand pendant, we manually moved the jaws so the light and X‐rays would be projected onto the active surface of the array. This corresponded to the following jaw settings X1: 1.4 cm, X2: 1.5 cm, Y1: 0.7 cm, Y2: 0.7 cm. We acquired images of light, 6 and 15 MV X‐rays with the above jaw settings at a collimator angle of 270° and then repeated these same measurements at a collimator angle of 90°. The signal received from the 15 MV X‐rays was smaller than the 6 MV X‐rays for the same delivered monitor units (MU). In order to compensate for this, the linear accelerator was programmed to deliver 3 MU for the 6 MV X‐rays and 6 MU for the 15 MV X‐rays. A brief outline of the theory that results in the energy dependence of the signal is provided in the Discussion section below.

### C. Jaw movement reproducibility

In order to test the reproducibility and precision of jaw movement, the following measurements were acquired with the device positioned in the same configuration as described in Section B. We first moved each jaw independently, using the hand pendent of the linear accelerator, such that the field size was projected onto the active portion of the detector at 100 cm SSD. After moving the asymmetric jaws to the above field size, we acquired an image of the light field produced using these jaw settings. After acquiring this first image, the jaws were opened to a larger field size. The second light field image was acquired after the jaws were returned to the original settings (X1: 1.4 cm, X2: 1.5 cm, Y1: 0.7 cm, Y2: 0.7 cm) using the linear accelerator's digital controls on the console. The third image was acquired after the jaws were again opened and again returned to the original field size using the computer console. In total, three light field images were acquired: one after the field size was set manually using the pendent, one after the field size was set using the linear accelerator console, and one after the field size was set a second time using the linear accelerator console.

### D. Analysis of edges

Radiation and light field edges were defined as the 50% intensity value within the penumbra region of the beam profile. To find the 50% point, each row of data was fitted to a sigmoidal curve using MATLAB's curve fitting toolbox (The MathWorks, Natick, MA). Each row was fit to the following mathematical equation:

(1) $$y=a+\frac{b-a}{1+e^{-(x-c)/d}}$$

The *c* parameter in the above equation corresponds to the 50% point. Parameters *a* and *b* are two asymptotic values of the function at large and small *x*. The *d* parameter is the width of the region of points between the two asymptotic values.^(15)^ Edges were determined by fitting the 50% point from each row to a straight line using linear regression. The line of best fit, defined by the y‐intercept and slope, is taken as the location of the field edge.

### E. Inter‐observer variability

In an effort to quantify the improvement over film and devices such as the PROFILER2, we had five colleagues perform radiation light congruence measurements on the same linear accelerator for identical setups, using the film technique, the PROFILER2, and the photodiode detector. The film technique had each observer mark the location of the light field edge using a pen and ruler such that the indentation created by the pen would be transferred onto the film. After marking the outside of the film jacket, the film was irradiated and then developed. Each observer compared the indentation created by the pen to the radiation field edge in order to determine the radiation light congruence. Using the PROFILER2, each observer set each of the jaws independently such that the light field corresponded to the 20 by 20 cm field size markings on the PROFILER2's surface at 100 SSD. After setting the jaws, the PROFILER2 was irradiated and the radiation light congruence for each observer was recorded. For the photodiode array, each individual setup and acquired measurement of radiation light congruence is described in Section B above.

## III. RESULTS

**Figure 2:**
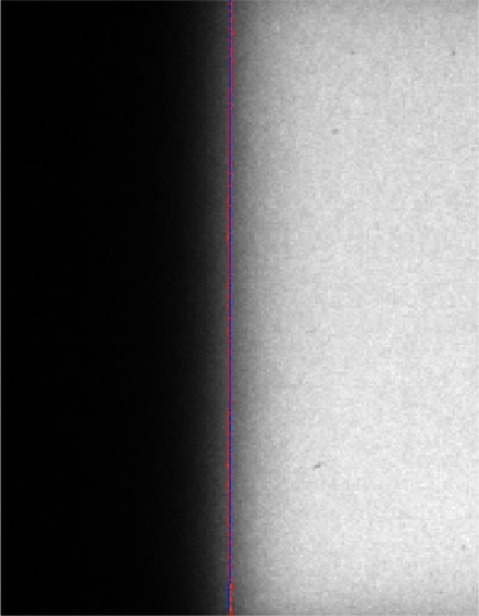
A representative radiation field image acquired using our device with the calculated field edge along each pixel row (red) and the resulting best fit field edge (blue).

**Figure 3:**
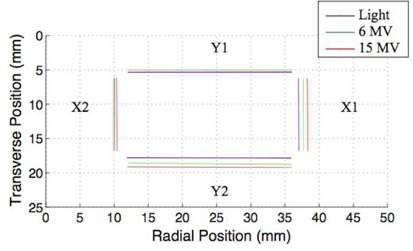
Edges of the light field, 6 MV X‐rays, and 15 MV X‐rays created by the asymmetric jaws (X1: 1.4 cm, X2: 1.5 cm, Y1: 0.7 cm, Y2: 0.7 cm) for collimator angle of 270°.

**Figure 4:**
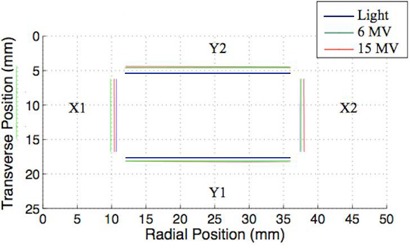
Edges of the light field, 6 MV X‐rays, and 15 MV X‐rays created by the asymmetric jaws (X1: 1.4 cm, X2: 1.5 cm, Y1: 0.7 cm, Y2: 0.7 cm) for collimator angle of 90°.

Figure 2 shows a representative radiation field edge image acquired using our device with the calculated field edge along each pixel row shown in red and the resulting best fit field edge shown in blue. Figures 3 and 4 and Tables 1 and 2 show the location of the edges of the light field, as well as the 6 and 15 MV X‐ray fields for collimator angles 270° and 90°. At a collimator angle of 270°, the radiation field edge differed from the light field edge by a maximum amount of 1.290±0.002 mm which was associated with the 15 MV X‐ray edge of the X1 jaw. Errors reported are standard errors. The smallest disagreement between the light field and radiation field was 0.016±0.003 mm which was associated with the 6 MV edge of the X2 jaw. At a collimator angle of 90°, the radiation field edge differed from the light field edge by a maximum amount of 0.932±0.003 mm which was associated with the 15 MV X‐ray edge of the Y2 jaw. The smallest disagreement between the light field and radiation field was 0.102±0.003 mm which was associated with the 6 MV edge of the X2 jaw. On the Varian Clinac 21EX linear accelerator, the light field gets reflected off a mirror which is attached to the collimator. As the collimator rotates, the mirror moves but the radiation source does not move. If the radiation or light sources are not on the rotational axis, this will result in a collimator angle dependence of the radiation light field congruence. The collimator angle dependence, in theory, could allow one to precisely determine the location of the X‐ray source.

**Table 1:** Radiation light congruence at collimator angle 270°.

*CA (degrees)*	*Edge*	*light/X‐ray*	*Energy (MV)*	*Edge (mm)*	δ *Edge (mm)*	*Slope*	δ *Slope*
		light	na	36.885	0.002	0.00031	0.00003
270	Right (X1 jaw)	X‐ray	6	37.718	0.002	0.00027	0.00002
		X‐ray	15	38.175	0.001	0.00037	0.00002
		light	na	10.053	0.003	0.00045	0.00004
270	Left (X2 jaw)	X‐ray	6	10.037	0.001	−0.00004	0.00002
		X‐ray	15	10.465	0.001	−0.00012	0.00002
		light	na	5.354	0.003	−0.00002	0.00002
270	Top (Y1 jaw)	X‐ray	6	5.032	0.002	−0.00005	0.00001
		X‐ray	15	5.315	0.002	−0.00006	0.00001
		light	na	17.860	0.003	0.00028	0.00001
270	Bottom (Y2 jaw)	X‐ray	6	18.688	0.001	0.00041	0.00001
		X‐ray	15	19.119	0.001	0.00041	0.00001

Edges of the light field, 6 MV X‐rays, and 15 MV X‐rays created by the asymmetric jaws (X1: 1.4 cm, X2: 1.5 cm, Y1: 0.7 cm, Y2: 0.7 cm) for collimator angle of 270°. The edges created by the y jaws are reported with respect to the top of the detector and the edges created by the x jaws are reported with respect to the left edge of the detector. Reported edges were from the middle of the observed edges.

**Table 2:** Radiation light congruence at collimator angle 90°.

*CA (degrees)*	*Edge*	*light/X‐ray*	*Energy (MV)*	*Edge (mm)*	δ *Edge (mm)*	*Slope*	δ *Slope*
		light	na	37.458	0.003	0.00097	0.00004
90	Right (X2 jaw)	X‐ray	6	37.560	0.001	0.00035	0.00002
		X‐ray	15	37.940	0.001	0.00047	0.00002
		light	na	10.638	0.002	0.00021	0.00002
90	Left (X1 jaw)	X‐ray	6	9.847	0.001	0.00005	0.00001
		X‐ray	15	10.356	0.001	0.00000	0.00002
		light	na	5.358	0.002	0.00017	0.00001
90	Top (Y2 jaw)	X‐ray	6	4.469	0.001	0.00007	0.00001
		X‐ray	15	4.426	0.002	0.00014	0.00001
		light	na	17.813	0.007	0.00039	0.00003
90	Bottom (Y1 jaw)	X‐ray	6	18.103	0.001	0.00051	0.00001
		X‐ray	15	18.185	0.002	0.00041	0.00001

Edges of the light field, 6 MV X‐rays, and 15 MV X‐rays created by the asymmetric jaws (X1: 1.4 cm, X2: 1.5 cm, Y1: 0.7 cm, Y2: 0.7 cm) for collimator angle of 90°. The edges created by the y jaws are reported with respect to the top of the detector and the edges created by the x jaws are reported with respect to the left edge of the detector. Reported edges were from the middle of the observed edges.

In general, for both collimator angles, the X2 jaw had better radiation light field congruence than the X1 jaw, and the Y1 jaw had better radiation light field congruence than the Y2 jaw.

These measurements were consistent with the radiation light field congruence measurements performed using the PROFILER2 system during the monthly quality control around the time of the measurements.

**Figure 5:**
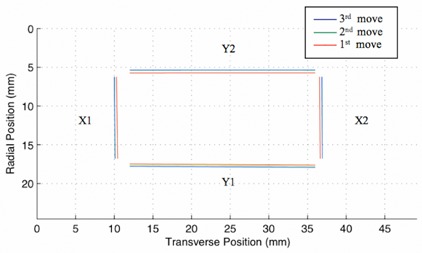
Edges of the light field created by the X and Y jaws. The red is the location of the light field edge after manually moving the jaws using the hand pendent of the linear accelerator. The blue line is the location of the light field edges after the jaws were first moved digitally using the linac console, and the green lines represent the field light edges after the second move using the console.

Figure 5 and Table 3 show the location of the light field edges for the jaw reproducibility test. The jaw reproducibility test found that moving the jaws with the console compared to the moving the jaws with the hand pendent resulted in an average discrepancy of 0.30±0.06 mm for each jaw. Also, the jaws returned to the same location to within 0.022±0.016 mm for the X1, X2, and Y1 jaws when using the console. The location of the Y2 jaw returned to the same location to within 0.138±0.006 mm when using the console. The location of each jaw is reported digitally by the linear accelerator to within 1 mm; however, we found that the linear accelerator controls the movement of the jaws to greater precision than 1 mm.

**Table 3:** Light field of jaw reproducibility tests.

*CA (degrees)*	*Edge*	*Light*	*Move*	*Edge (mm)*	δ *Edge*	*Slope*	δ *Slope*
		light	1st move	36.600	0.003	0.00039	0.00004
270	Right (X1 jaw)	light	2nd move	36.888	0.002	0.00034	0.00003
		light	3rd move	36.885	0.002	0.00031	0.00003
		light	1st move	10.370	0.003	0.00069	0.00004
270	Left (X2 jaw)	light	2nd move	10.084	0.003	0.00045	0.00004
		light	3rd move	10.053	0.003	0.00045	0.00004
		light	1st move	5.719	0.004	−0.00002	0.00002
270	Top (Y1 jaw)	light	2nd move	5.386	0.004	0.00002	0.00002
		light	3rd move	5.354	0.003	−0.00002	0.00002
		light	1st move	17.545	0.003	0.00029	0.00001
270	Bottom (Y2 jaw)	light	2nd move	17.722	0.003	0.00028	0.00001
		light	3rd move	17.860	0.003	0.00028	0.00001

Edges of the light field created by the x and y jaws. The “1st move” is the location of the light field edge after manually moving the jaws using the hand pendent of the linear accelerator. The “2nd move” is the location of the light field edges after the jaws were first moved digitally using the computer console, and the “3rd move” represents the field light edges after the second move using the console. The edges created by the y jaws are reported with respect to the top of the detector and the edges created by the x jaws are reported with respect to the left edge of the detector.

The standard deviation between interobserver radiation light congruence measurements using film was 0.3 mm, the standard deviation between the interobserver measurements using the PROFILER2 was 0.5 mm, and for the photodiode detector the standard deviation between repeated radiation light measurements was 0.08 mm (see Table 4).

**Table 4:** Mean of radiation light field congruence and standard deviation of interobserver variation using film, PROFILER2, and our device.

*Method*	*Mean (mm)*	*Standard Deviation (mm)*
Film	0.6	0.3
PROFILER2	0.4	0.5
Device	1.13	0.08

## IV. DISCUSSION

Though film and devices such as the PROFILER2 are adequate for routine checks, both of these measurements are highly user‐dependent as they rely on the user's interpretation of the field edges for both light and X‐ray fields. The PROFILER2 does not directly measure the profile of the light field – it measures only the profile of the radiation field. Radiation light field congruence is then calculated by comparing the unmeasured light edges with the measured radiation edge. The PROFILER2 assumes that when analyzing the field edges, the light field edges are aligned with one of the rectangular field marks on the surface of the detector.^(16)^

The main advantage of the photodiode detector described in the current study is that it eliminates interobserver variability by automating measurements. The reported standard deviation between interobserver measurements demonstrates significant improvement in consistency of measurements between observers over both film and PROFILER2 measurements. The ability to make measurements with very low interobserver variability should prove useful during the commissioning of new linear accelerators because it sets an objective “base line” with which to compare future measurements. Reduced variability also improves monthly and annual quality control measurements and allows for more accurate evaluation of trends, providing insight into potential issues sooner than user‐dependent quality control procedures. For example, this would allow medical physicists to identify a drifting machine parameter before it becomes a problem clinically.

Another advantage of the photodiode device is its ability to determine the relative position of the light field edge with submillimeter accuracy. This level of accuracy goes well beyond that required for monthly or annual quality control, but it would prove useful for calibrating the primary jaw positions, since the accuracy of that procedure relies on the accuracy of the light field. Jaw calibration is done by projecting the light field onto a surface with known position markings. The jaws are moved until the edge of field light corresponds with a specific marking on the external surface and that jaw coordinate is recorded. Using a photodiode array for this calibration would allow for precise movements of the jaws from one position to the next, resulting in improved calibration accuracy. In addition to using the device for the calibration of the jaws, a simple subtraction of two images of an MLC leaf or set of MLC leaves in two different orientations could be used to get an independent check of the position of the MLC. The primary disadvantage of the photodiode device is the small active area (2.5 cm by 5 cm) of the detector, which limits its use to measuring small field sizes or small sections of larger field sizes. Rad‐icon Imaging Corporation does produce larger active area detectors (up to 10 by 10 cm) but these larger detectors are significantly more expensive. Off the shelf, the detector is optimized for detection of kV X‐rays. As observed, the device generates a larger signal for 6 MV than for 15 MV photon beams for the same number of delivered MUs. Further, the addition of a buildup layer significantly improves the detection of MV X‐rays for both 6 MV and 15 MV X‐rays. The different response to photon energies can be explained by the observation that the weighted average interaction cross section for the range of photon energies present in a 6 MV photon beam is larger than the weighted average photon interaction cross section for the range of energies present in a 15 MV photon beam, for both the buildup layer and the scintillator. The enhanced photon response with the addition of the buildup layer can be explained by the fact that photons interact within the buildup region resulting in additional electrons becoming liberated from the acrylic plastic, which then are able to cause additional ionization events within the scintillating material. Electrons scattering from the buildup layer will cause blurring of the field edge, but this will have a minimal effect on our calculated field edge due to the isotropic scattering of the electrons. In general, a larger number of photon interactions result in a larger number of liberated electrons, predominantly through Compton interactions, which result in a greater amount of energy deposited in the scintillator, and a larger scintillation response. As a result, the 15 MV photon beams produce weaker scintillation signals than the 6 MV photon beams, and adding 1.5 mm of acrylic buildup enhances the scintillation signal for both energies for the same number of delivered MUs.

Future work could look at optimizing the thicknesses of the buildup layer and scintillation layers based on interaction cross sections and electron stopping powers. Also, the measurements made using the device could be used to precisely determine the location of the X‐ray source and light source.

## V. CONCLUSIONS

The photodiode detector offers an improved method of measuring geometric machine parameters, as demonstrated by measurements of radiation light congruence and jaw movement reproducibility. We have shown that the device is capable of measuring these geometric machine parameters to greater accuracy levels and with greater consistency between observers than other available methods. It is anticipated that this device will also increase consistency over time. We expect the device to find use in various settings, including the commissioning of new linear accelerators, monthly and annual quality control measurements, and stereotactic radiosurgery.
